# Germline mutations as potential causes of childhood solid tumours: comments on the Norwegian childhood cancer cohort study

**DOI:** 10.1038/s41416-018-0059-0

**Published:** 2018-03-29

**Authors:** Yaddanapudi Ravindranath, Logan G Spector

**Affiliations:** 1 Wayne State University School of Medicine, Division of Hematology/Oncology, Children’s Hospital of Michigan, 3901 Beaubien Boulevard, Detroit, MI 48201 USA; 2 Department of Pediatrics, University of Minnesota, 420 Delaware Street, MMC 715, Minneapolis, MN 55455 USA

## Abstract

Some cancer predisposing germline mutations cause overt birth defects and congenital anomalies. Others are clinically silent and can only be suspected by the presence of increased cancer incidence in family members. A new study shows that long-term monitoring of families may be needed to discover previously unsuspected underlying cancer predisposing mutations.

The diagnosis of cancer in children has a life-changing impact on the families involved. Two questions often posed by the families are: how did my child get cancer, and is there a cure for it? Although we are moving closer to curing childhood cancer than ever before, the identification of causative risk factors continues to be elusive for the vast majority of these cancers. The definition of the fundamental pathways involved in cancer initiation and propagation,^1^ nearly two decades ago, led to a paradigm shift in the search for causative factors. As a result, the hunt has now shifted from searching for non-genetic causes such as environmental exposures, to genetic causes, including predisposing mutations, modifier (epigenetic) mutations, and oncogenic driver mutations. This search has, however, proven difficult in children due to the low frequency of childhood cancer and the multiplicity of cancer types. In addition, past studies have shown that clinically definable cancer predisposition syndromes account for <10% of childhood cancer cases.^2,3^

In a study reported in the *British Journal of Cancer*, Kollerud et al. sought to identify familial risk factors for the occurrence of apparently ‘sporadic’ childhood solid tumours.^4^ This cancer cohort study, which used data from the Norwegian Family and Life Course Study and from the Norwegian Cancer Register, included 2,610,937 children born between 1960 and 2001. During this period, 2477 primary childhood solid tumours (except lymphoma) were diagnosed, and the authors assessed the risk of cancer occurrence against a history of solid cancer occurrence in the immediate family. The study shows that intra-family risk persists, even after the exclusion of known hereditary cancer syndromes, for three tumour types—neuroblastoma, hepatoblastoma, and melanoma. This observation is of particular interest for paediatric melanoma, given that distinction of benign Spitz nevus versus true malignant melanoma is often difficult to distinguish histologically. Therefore, a family history of cancer in such cases should increase the suspicion of a true malignant melanoma, and newer molecular profiling of the tumour should be undertaken, as is now standard practice in adult melanoma clinics.

Another interesting observation of this study was the association of solid tumour risk with structural birth defects; 131/2477 or 5.2% of children with solid tumours had birth defects and neural tube defects, and these were more common in children with CNS tumours. Of note, incidence of the Li-Fraumeni cancer predisposition syndrome, identified using Chompret’s criteria,^5^ was 2%, and highest in CNS tumours; these findings are remarkably close to the prevalence identified in the earlier study by Zhang et al.^3^ The 5.2% incidence of birth defects reported here is likely an underestimate, given that this is likely derived from incidental findings on clinical examination rather than active surveillance. Children with cancer now undergo multiple imaging studies, and this provides an opportunity for additional birth defects to be identified, such as ostium secundum defects on echocardiograms,^6,7^ bone defects (e.g., including bone cysts, hemi vertebrae, rib anomalies) and other defects (including renal anomalies, renal cysts, ectopic kidney, Horseshoe kidney and hamartomas), on radio imaging and computerised tomography. However, further research linking the risk of specific cancers to specific birth defects remains to be completed.^8^

Although this study took place within the ethnically homogenous and closely-tracked Norwegian population, we expect that similar results would apply elsewhere. The results of this study stress the need for a more careful clinical documentation of the history of cancer within a family, particularly among first-degree relatives. It must also be firmly recognised that some of these occurrences might happen several years after diagnosis in the index case. This study largely replicates what is now known about the frequency of familial syndromes; however, the continued elevation in risk after controlling for these suggests there are new syndromes to be discovered. Some syndromes have only recently become clear, including ETV6, PAX5 and RUNX1 germline mutation syndromes,^9,10^ and some birth defect/cancer syndromes have only recently been hinted at (e.g., GU anomalies and hepatoblastoma).^11^ Birth defects/dysmorphology on the other hand may point to mutations in cancer predisposition pathways, such as the rib anomalies that occur in Gorlin syndrome.^7^ For this reason, we may wish to concentrate the search for additional predisposition genes to familial clusters or cases with congenital anomalies.
